# Short-term changes in nightlife attendance and patron intoxication following alcohol restrictions in Queensland, Australia

**DOI:** 10.1186/s12889-018-6098-x

**Published:** 2018-11-12

**Authors:** Kerri Coomber, Renee Zahnow, Jason Ferris, Nicolas Droste, Richelle Mayshak, Ashlee Curtis, Kypros Kypri, Dominique de Andrade, Kristy Grant, Tanya Chikritzhs, Robin Room, Heng Jiang, Nicholas Taylor, Jake Najman, Peter Miller

**Affiliations:** 1 School of Psychology, Deakin University, Geelong, Australia; 2 School of Social Science, The University of Queensland, St Lucia, Australia; 3 Centre for Health Services Research, The University of Queensland, Woolloongabba, Australia; 4 School of Medicine and Public Health, University of Newcastle, Callaghan, Australia; 5 Lives Lived Well Research Group, School of Psychology, The University of Queensland, St Lucia, Australia; 6 Australian Institute of Tropical Health & Medicine, James Cook University, Cairns, Australia; 7 National Drug Research Institute, Curtin University of Technology, Bentley, Australia; 8 Centre for Alcohol Policy Research, La Trobe University, Bundoora, Australia; 9 Centre for Social Research on Alcohol and Drugs, Stockholm University, Stockholm, Sweden; 10 Queensland Alcohol and Drug Research and Education Centre, School of Public Health, University of Queensland, St Lucia, Australia

**Keywords:** Alcohol, Policy, Patron interviews, Nightlife, Intoxication

## Abstract

**Background:**

This study aims to explore short-term changes following the introduction of alcohol restrictions (most notably 2 am to 3 am last drinks). We examined patterns of nightlife attendance, intoxication, and alcohol use among patrons shortly before and after restrictions were introduced in Fortitude Valley, Brisbane: the largest night-time entertainment precinct of Queensland.

**Methods:**

Street-intercept patron interviews were conducted in Fortitude Valley in June (*n* = 497) and July (*n* = 562) 2016. A pre-post design was used to assess changes in time spent out drinking/partying prior to the interview, time of arrival in the precinct, pre-drinking, and blood alcohol concentration (BAC).

**Results:**

Regression models indicated that after the policy introduction, the proportion of people arriving at Fortitude Valley before 10:00 pm increased (OR = 1.38; 95% CI = 1.04, 1.82). Participants reported going out, on average, one hour earlier after the intervention (β = − 0.17; 95% CI = 0.11, 0.22). There was a decrease (RRR = 0.58; 95% CI = 0.43, 0.79) in the proportion of participants who had a high level of intoxication (BAC ≥0.10 g/dL) post-intervention. No other significant differences were found.

**Conclusions:**

Earlier cessation of alcohol sales and stopping the sale of rapid intoxication drinks after midnight was associated with people arriving in Fortitude Valley earlier. Though legislative loopholes allowed some venues to continue trading to 5 am, the proportion of people in the precinct who were highly intoxicated decreased after the restriction. Further measurement will be required to determine whether the reduction has persisted.

## Background

Restricting the hours alcohol can be sold is an effective and inexpensive way of reducing alcohol-related assaults and unintentional injury [1, 2]. In 2008, a multi-component strategy including the restriction of trading hours from 5 am to 3:30 am in Newcastle, New South Wales, was followed by a 37% decrease in assaults compared to a control site, and an average reduction of 344 emergency department attendances per year [3, 4]. When last analysed [3, 5], this downward trend in assaults had been sustained for eight years. Conversely, increased venue trading hours in Perth, Western Australia, was associated with a 70% and 47% increase in the incidence of assault and drink-driving, respectively [6, 7]. International research also demonstrates an increased rate of alcohol-related assault and unintentional injury after extending venue trading hours [8, 9].

In February 2016, the Queensland state government passed legislation based on the Newcastle model. Coming into effect on 1 July 2016 (see http://www.webcitation.org/6rXxbYEe8 for full details), the multi-faceted Queensland Tackling Alcohol-Fuelled Violence Policy required the cessation of alcohol service (‘last drinks’) by 3 am within defined entertainment areas (Safe Night Precincts) and by 2 am in the rest of the state. However, venues could apply for an extended trading permit allowing sales of alcohol until 5 am; venues could apply for up to 12 single night permits within a 12-month period. A state-wide ban on the service of ‘rapid intoxication drinks’ (e.g., shots) after midnight was also introduced.

In this study we undertook patron interviews in the entertainment precinct of Fortitude Valley, Brisbane, in the month before and after the introduction of the policy, to test for short-term changes in drinking behaviour.

## Methods

### Sample

Interviews were conducted from 11:00 pm-5:59 am on three Saturday night/Sunday mornings before the change (11/12 June, 18/19 June, and 25/26 June 2016) and four Saturday night/Sunday mornings after the change (2/3 July, 8/9 July, 23/24 July, and 30/31 July 2016). Following established protocols [10, 11], every third person passing the research teams (who were wearing easily identifiable institutional clothing) was invited to participate. Participants who provided verbal consent were given a business card with study and contact details.

### Measures

#### Demographics and current night

Participants’ gender and age were recorded in the following categories (18–20 years; 21–25 years; and > 25 years) [12]. Participants were asked how long they had been out and what time they arrived in the precinct. Based on the distribution of responses, a cut-point of arrival up to 10:00 pm versus after 10:00 pm was used.

#### Alcohol use

Participants were asked how many standard drinks they consumed prior to entering a licensed venue (pre-drinking). Participant alcohol status at the time of interview was measured, after confirming that at least 10 min had elapsed since eating or drinking, using a breathalyser (Andatech Prodigy S, calibrated six-monthly) that converted breath alcohol level into an estimate of blood alcohol concentration (BAC). BAC was examined as both a continuous measure and an ordinal categorical measure in g/dL. The BAC categories used were: ‘sober’ (0.0); ‘slightly intoxicated’ (> 0.0 to < 0.05); ‘moderately intoxicated’ (≥0.05 to < 0.10); and ‘highly intoxicated’ (≥0.10). Our research indicates that observers of licensed venue patrons are able to reliably estimate level of intoxication based on physical signs, with interviewer-rated intoxication moderately correlated with BAC [13]. Therefore, we use the terms BAC and intoxication interchangeably throughout the paper.

### Procedure

**Fig. 1:**
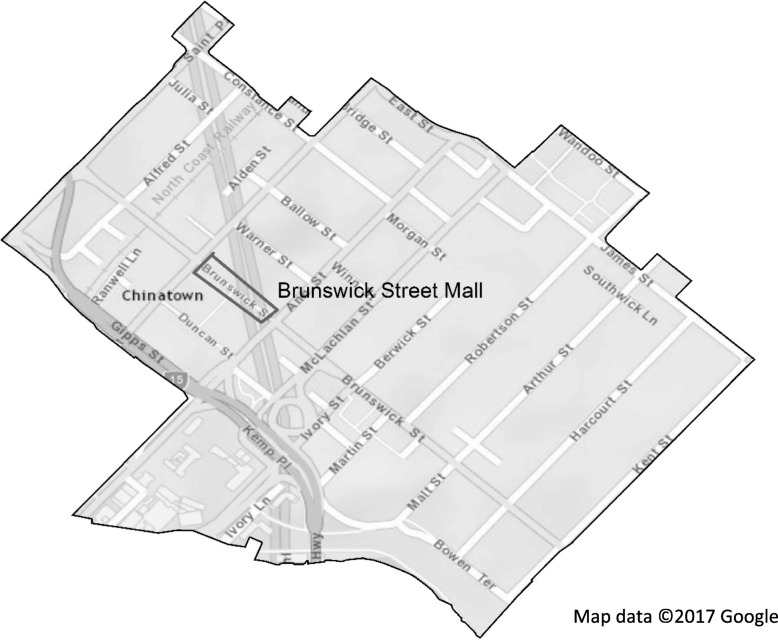
Boundary of Fortitude Valley entertainment precinct and location of interviewing (Brunswick Street Mall). Map data: Google, DigitalGlobe

Ethical approval was obtained from Deakin University (2011–095) and The University of Queensland (20160010121). Five-minute interviews were conducted with patrons in Brunswick Street Mall, the main thoroughfare of Fortitude Valley (see Fig. 1), using a mobile survey application (TapForms™) on an iPod Touch.

### Analysis

Linear, logistic, and negative binomial regression models examined differences in participant responses by month of interview, with hour of interview as a covariate. For the ordinal BAC variable, ordered logistic regression was planned. However, the assumption of parallelism was not met (Brant test for month = 13.18, *p* = 0.001). Therefore, three multinomial logistic regression models were conducted to explore potential BAC change among those who had a BAC greater than zero; the BAC categories of slight, moderate, and, high intoxication were each used as the reference category. This approach was used to provide insight into how patrons shifted in their levels of intoxication. All analyses were conducted using Stata 14.0.

## Results

### Demographics

Research teams approached 1267 people, of whom 1059 completed the interview (June *n* = 497; July *n* = 562), with an overall response rate of 84% (June 83%; July 85%). For the June sample, participant mean age was 23 years (standard deviation (SD) = 5.9), and 65% were male. In the July sample, the mean age was 24 years (SD = 6.6), and 62% were male. There were no significant differences in age and gender distribution by month. Seventy-three participants (7%) refused the breath-test (June *n* = 50, 10%; July *n* = 23, 4%).

### Hours spent out and time of arrival at precinct

Participants interviewed in July started their night earlier, relative to their interview time, than those interviewed in June (see Table 1). Further, a significantly smaller proportion of participants arrived in Fortitude Valley after 10:00 pm in July, compared to June.

**Table 1:** Regression models testing change in outcome variables pre- and post-intervention

Variable	June M (SE) or %	July M (SE) or %	Test statistic	95% CI
Hours out (*n* = 1058)	4.6 (0.14)	5.7 (0.13)	β = 0.17	0.11, 0.22
Arrival at precinct ≤10:00 pm (*n* = 900)	43%	50%	OR = 1.38	1.04, 1.82
Pre-drinking (yes) (*n* = 1035)	85%	85%	OR = 1.05	0.75, 1.48
Number of pre-drinks (*n* = 879)	8.1 (0.28)	7.5 (0.25)	IRR = 0.93	0.84, 1.02
Mean BAC (g/dL) (*n* = 986)	0.081 (0.033)	0.079 (0.002)	β = −0.003	−0.010, 0.005
BAC categories (g/dL) (n = 986)				
0.0 (sober)	15%	12%	RRR = 0.60	0.40, 0.90
> 0.0 to < 0.05 (slight)	17%	17%	RRR = 0.73	0.50, 1.06
≥ 0.05 to < 0.10 (moderate)	30%	41%	RRR = 1.00	
≥ 0.10 (high)^a^	38%	30%	RRR = 0.58	0.43, 0.79

Note. All models adjust for hour of interview. June is the reference category for all models

^a^Within the BAC category of ≥0.10 g/dL, 74% of participants in both June and July recorded a BAC of ≥0.12 g/dL and 28% (June) to 32% (July) had a BAC of ≥0.15 g/dL

M = Mean

SE = Standard error

95% CI = 95% confidence interval

β = Beta-weight

OR = Odds ratio

IRR = Incidence rate ratio

RRR = Relative risk ratio

### Pre-drinking and blood alcohol concentration (BAC)

There was no significant difference in the proportion of participants pre-drinking, or the number of standard drinks consumed while pre-drinking, in July versus June (Table 1).

There was no significant difference in the overall mean BAC of participants interviewed in July compared to June. Compared to slight intoxication, there were no significant shifts in the proportions of patrons in the higher BAC categories. However, compared to moderate intoxication, there was a decrease in the proportion of participants who were highly intoxicated in July compared to June and a small, but significant, decrease in those with a zero BAC (see Table 1). There was a corresponding increase in the proportion of participants who recorded a moderate intoxication in July versus June (reference category of ≥0.10 g/dL; RRR = 1.73, 95% confidence interval = 1.27, 2.35). No other significant differences were found.

## Discussion

In the month following the introduction of new legislation on alcohol restrictions in Queensland, a smaller proportion of patrons in the Fortitude Valley precinct had a BAC ≥0.10 g/dL than in the pre-change period. A corresponding shift in the proportion of patrons to lower BACs (from a BAC of ≥0.10 g/dL to a BAC of ≥0.05 to < 0.10 g/dL) was also observed in the post-change period. While it appears the majority of patrons still consume alcohol to moderate intoxication, the finding of a reduction in the number of highly intoxicated patrons is encouraging. This change in patterns of intoxication coincided with a larger proportion of patrons arriving before 10:00 pm and patrons commencing their evenings out an hour earlier.

The finding that people went out earlier after the restriction on last drinks aligns with previous cross-sectional research into the trading hours restrictions implemented in Newcastle [14]. Our findings suggest that patrons responded to the policy changes by starting to socialise, and arriving in the precinct earlier [14]. Despite patrons having spent more time out and more time within the Fortitude Valley precinct after the policy introduction, there was a decline in the proportion of highly intoxicated patrons. This reduction in percentage of highly intoxicated patrons may be partially driven by the ban on rapid intoxication drinks after midnight. Further research is needed to explore both the drivers of these changes in consumption patterns and whether the changes were sustained over time. Encouragingly, there was no significant increase in the prevalence of pre-drinking.

While the findings indicate some changes in patterns of drinking and intoxication immediately after the introduction of the alcohol restrictions, the use of extended trading permits allowing venues to continue serving alcohol until 5 am may have reduced the impact of earlier last drinks [15, 16]. While the earlier last drinks had the potential to reduce alcohol service across 34 venues in Fortitude Valley by 68 h each night, there was no weekend in July 2016 (after the restriction) in which all venues stopped serving alcohol at 3 am [15, 16]. The objective of the new legislation was undermined by the continued provision of these extended trading permits.

### Limitations

This preliminary study compared patterns of drinking and intoxication before and after new alcohol restrictions in a major entertainment precinct in Queensland. The inclusion of control sites was impracticable given the short lead-time between funding of the research and the legislation coming into effect. Accordingly, the shortcomings of pre-post designs limit what can be inferred from the observed changes.

The primary threat to validity is competing interventions or conditions that exert downward pressure on socialising behaviour and drinking, for instance, changes in economic or climatic conditions. Neither seems likely given the short timeframe of the pre- and post-measurement periods -- a total period of less than two winter months. A secondary limitation is that the university semester ended in late June. This may have resulted in differences between the pre- and post-intervention populations, because fewer students were going out in June because of examinations, or because students from outside of Brisbane returned home during the between-semester break. Our interview did not capture information that would allow us to identify students and adjust for differences in the distributions. However, we found no significant difference in age (June M = 23 years; July M = 24 years) or gender (June = 65% male; July = 62% male) distribution between the June and July samples. As far as we are aware, there were no other legislative or policy changes at the national, state, or local levels that could account for the observed changes in this period.

Such considerations will come into play in the evaluation of longer-term changes. We are currently collecting patron interview data in additional precincts of Queensland. This will allow us to examine longer term trends in drinking and intoxication in Safe Night Precincts and other areas, including asking patrons if, and how, they have changed their behaviour on their nights out since the introduction of the policy [see [17]]. Based on observations in Newcastle, we expect to see reductions in intoxication and harm as licensees modify their business practices in response to the new conditions [14]. A strength of this preliminary evaluation and the ongoing work is the objective measurement of BAC afforded by the use of breathalysers and the sampling protocol we employed.

Despite the high response rate of 84%, it is possible the participants in this study may not be fully representative of all people who attend night-time entertainment precincts; however, we suggest the findings would generalise to a large proportion of the population exposed to these areas.

## Conclusions

The restrictions implemented in Queensland were followed by an immediate reduction in the prevalence of highly intoxicated patrons in Fortitude Valley, although average BAC levels did not change. This happened despite the widespread use of extended trading permits, which undermined the objective of the legislation. Further research is necessary to determine how drinking patterns change in the longer term and what occurs in other parts of the state.

## Abbreviations

BAC: Blood alcohol concentration
CI: Confidence interval
IRR: Incidence rate ratio
M: Mean
OR: Odds ratio
RRR: Relative risk ratio
SD: Standard deviation
SE: Standard error
